# Influencing factors and risk prediction nomogram model construction of hyperkalemia during kidney transplantation

**DOI:** 10.3389/fsurg.2025.1657585

**Published:** 2026-02-12

**Authors:** Kun Dong, Guanmiao Chen, Yongyuan Jian, Ruiling Su, Junze Chen, Cheng Zhang, Kaiyong Huang, Xuelin Tan, Bo Peng, Ping Huang, Chunqiang Dong, Hongwei Yang

**Affiliations:** 1 Dalian Medical University, Dalian, China; 2 Department of Organ Transplantation, The First Affiliated Hospital of Guangxi Medical University, Nanning, Guangxi, China; 3 Organ Transplant Center, Northern Theater Command General Hospital, Shenyang, China

**Keywords:** hyperkalaemia, kidney transplantation, risk factors, nomogram, predictive model

## Abstract

**Objective:**

To analyze the risk factors of hyperkalemia during kidney transplantation, and to construct the prediction model of nomogram.

**Methods:**

162 cases of renal transplant patients in our hospital from January 2020 to September 2024 were included. The clinical data of the patients were retrospectively analyzed. According to whether hyperkalemia occurred during the operation, the patients were divided into non hyperkalemia group and hyperkalemia group. The related factors of hyperkalemia in renal transplant patients were analyzed by multivariate logistic regression, and the nomogram model was constructed.

**Results:**

Among 162 renal transplant patients, 59 cases (36.42%) had high potassium during operation. Univariate analysis showed that the pre-operative blood potassium, pulse pressure, and hemodialysis time of the high potassium group were higher than those of the non high potassium group, and the pH value of the high potassium group was lower than that of the non high potassium group, the difference was statistically significant (*P* < 0.05). The results of logistic regression analysis showed that high preoperative blood potassium, low preoperative pH value, large pulse pressure, and long hemodialysis time were risk factors for Hyperkalemia during kidney transplantation surgery (*P* < 0.05). The area under the ROC curve for the training set and validation set of the nomogram model constructed based on the aforementioned risk factors was 0.933 (95% CI: 0.885-0.981, *P* < 0.05), 0.798 (95% CI: 0.662–0.935, *P* < 0.05). The sensitivity, specificity, logit value, and cutoff value were 0.892, 0.872, −0.625, 0.6 and 0.862, 0.700, −0.159, 0.5, respectively. The calibration curve and decision curve results indicate that the model has high predictive performance and clinical application value.

**Conclusions:**

High preoperative serum potassium, low preoperative pH, high pulse pressure, and long hemodialysis time are the risk factors of hyperkalemia in renal transplantation. According to the risk factors, constructing nomogram model to predict hyperkalemia in renal transplantation has high clinical value.

## 1 Introduction

Hyperkalemia is primarily attributed to potassium redistribution from intracellular compartments or impaired renal potassium excretion. Mild cases may present with nausea, diarrhea, and muscle weakness in patients, whereas severe cases can lead to arrhythmias, cardiac arrest, and sudden death (1). Among hemodialysis patients, the mortality rate increases by 4.2%, 11.1%, 16.6%, 26.6%, and 31.7% when blood potassium levels are <5.0, 5.0–5.5, 5.5–6.0, 6.0–6.5, and >6.5 mmol/L, respectively (2). Clinical observations indicate that some patients experience elevated blood potassium levels during kidney transplantation. Without timely intervention informed by extensive clinical experience, this condition can result in severe complications. Hyperkalemia is associated with renal failure, restricted renal potassium excretion, and pharmacological treatments (3); however, the precise mechanisms underlying hyperkalemia during kidney transplantation remain unclear. Therefore, identifying influencing factors and constructing a predictive nomogram model for hyperkalemia during kidney transplantation could facilitate early intervention and significantly improve patient outcomes. This study aims to analyze risk factors for hyperkalemia during kidney transplantation and construct a predictive model to provide a foundation for enhancing treatment efficacy.

## 2 Materials and methods

### 2.1 Study subjects

A retrospective analysis was conducted on the clinical data of patients who underwent kidney transplantation at our hospital between January 2020 and September 2024. The intraoperative fluid replacement volume for all patients did not exceed 250 ml of 0.9% Rindl solution (the effect on potassium ion concentration was negligible). The immunization regiments for all patients were tacrolimus capsules (Hangzhou Zhongmei Huadong Pharmaceutical Co., LTD.) + mycophenolate mofetil enteric-coated tablets (Chengdu Shengdi Pharmaceutical Co., LTD.) + methylprednisolone sodium succinate for injection (Hanhui Pharmaceutical Co., LTD.). Inclusion criteria were as follows: (1) End-stage renal disease meeting the indications for kidney transplantation; (2) Age ≥18 years. Exclusion criteria included: (1) Multiple organ transplantation; (2) Secondary transplantation; (3) Incomplete data due to various reasons; (4) Preoperative blood potassium >5.0 mmol/L.

### 2.2 Data collection

The following data were collected: patient gender, age, past medical history (presence or absence of hypertension and diabetes), preoperative blood potassium, sodium, calcium, phosphorus, magnesium, creatinine, bicarbonate ion (HCO3-), albumin, glomerular filtration rate, hemoglobin, blood glucose, pH value, total cholesterol, use of angiotensin-converting enzyme inhibitors/angiotensin receptor blockers (ACEI/ARB), pulse pressure, and hemodialysis duration.

### 2.3 Data availability and handling of missing data

This was a retrospective study based on electronic medical records. Patients with any missing data in the key variables pre-defined for analysis (e.g., preoperative serum potassium, donor type, cold ischemia time, etc.) were excluded as per the exclusion criteria. Among the initially screened 178 patients, 16 (8.99%) were excluded primarily due to incomplete archival records. Given the low percentage of missingness and the lack of evidence suggesting a non-random pattern, the complete-case analysis employed here was considered statistically appropriate and was necessary for the integrity of the nomogram construction.

Variable selection was based on prior literature (2–4) and clinical expertise: indicators known to be associated with potassium balance (e.g., preoperative serum potassium, pH, hemodialysis duration) and renal hemodynamics were included. However, due to the retrospective nature of the study, some potential variables (e.g., detailed records of diuretic use, severity of cardiovascular comorbidities such as heart failure) were not consistently documented in electronic medical records and thus excluded, which may have impacted the model's comprehensiveness.

### 2.4 Diagnosis criteria and grouping of hyperkalemia

Recent clinical guidelines suggest lowering the threshold for diagnosing hyperkalemia. According to the latest “Expert Consensus on the Management of Blood Potassium in Chinese Patients with Chronic Kidney Disease” (4), blood potassium >5.0 mmol/L is considered diagnostic for hyperkalemia. Patients with blood potassium >5.0 mmol/L during kidney transplantation were categorized into the hyperkalemia group, while others were classified as the non-hyperkalemia group.

### 2.5 Construction and validation of the nomogram

Using the RMS package in R4.3.2 software, a nomogram model was constructed to predict hyperkalemia during kidney transplantation. The dataset was randomly divided into a training set (*n* = 113) and a validation set (*n* = 49) at a 7:3 ratio. Model performance was assessed using the receiver operating characteristic (ROC) curve. The Hosmer-Lemeshow goodness-of-fit test and calibration curve were employed to evaluate model fit and calibration. Decision curve analysis was used to assess the clinical net benefit of the model. Calibration was further validated using the Bootstrap algorithm with 500 resamples.

### 2.6 Statistical methods

Statistical analyses were performed using SPSS 25.0 software. Normally distributed measurement data were expressed as mean ± standard deviation, and intergroup comparisons were conducted using the t-test. Count data were presented as percentages, and intergroup comparisons were analyzed using the chi-square test. Multivariate logistic regression was utilized to identify risk factors for hyperkalemia during kidney transplantation. The nomogram model for predicting hyperkalemia during kidney transplantation was constructed and validated using R 4.3.2 software. A *p*-value < 0.05 was considered statistically significant.

## 3 Results

### 3.1 Univariate analysis of hyperkalemia during kidney transplantation surgery

Among 162 kidney transplant patients, 59 cases (36.42%) had high potassium levels during surgery. The preoperative blood potassium, pulse pressure, and hemodialysis time of the high potassium group were higher than those of the non high potassium group. The pH value of the high potassium group was lower than that of the non high potassium group, and the difference was statistically significant (*P* < 0.05), as shown in Table 1.

**Table 1:** Univariate analysis of hyperkalemia during kidney transplantation [*n* (%)/($\bar{x}\pm s$)].

Variable	Hyperkalemia group (*n* = 59)	Non-hyperkalemia group (*n* = 103)	*χ^2^/t*	*P*
Gender (Male/female)	32/27	55/48	0.011	0.918
Age (years)	36.53 ± 11.20	35.67 ± 10.37	0.491	0.451
BMI (kg/m^2^)	20.24 ± 1.95	20.07 ± 2.23	0.486	0.370
Combined hypertension	17 (28.81)	23 (22.33)	0.848	0.357
Combined diabetes	13 (22.03)	18 (17.48)	0.504	0.478
Preoperative blood potassium (mmol/L)	4.48 ± 0.18	4.34 ± 0.17	5.220	<0.001
Preoperative blood sodium (mmol/L)	134.42 ± 6.41	136.46 ± 6.28	−1.984	0.887
Preoperative blood calcium (mmol/L)	2.47 ± 0.29	2.55 ± 0.30	−1.646	0.784
Preoperative blood phosphorus (mmol/L)	2.57 ± 0.15	2.51 ± 0.17	2.022	0.284
Preoperative blood magnesium (mmol/L)	1.19 ± 0.14	1.17 ± 0.11	1.043	0.260
Preoperative creatinine (*μ*mol/L)	527.12 ± 145.83	517.35 ± 139.87	0.421	0.294
HCO_3_^−^(mmol/L)	17.27 ± 3.21	17.59 ± 3.07	−0.629	0.390
Preoperative albumin (g/L)	38.26 ± 3.09	39.31 ± 2.97	−2.134	0.845
Preoperative glomerular filtration rate (mL/min)	8.06 ± 2.04	8.22 ± 2.15	−0.483	0.882
Preoperative hemoglobin (g/L)	107.92 ± 9.99	104.25 ± 10.03	2.246	0.845
Preoperative blood glucose (mmol/L)	5.24 ± 1.18	5.36 ± 1.07	−0.639	0.739
Preoperative pH	7.34 ± 0.06	7.37 ± 0.05	−3.923	<0.001
Total cholesterol (mmol/L)	5.13 ± 1.03	4.88 ± 1.08	1.430	0.317
Take ACEI/ARB	20 (33.90)	21 (20.39)	3.622	0.057
pulse pressure (mmHg)	48.75 ± 9.41	42.16 ± 5.29	4.952	<0.001
Hemodialysis time (years)	3.26 ± 0.68	2.79 ± 0.59	4.326	<0.001

### 3.2 Multivariate logistic regression analysis of hyperkalemia occurring during renal transplantation

Taking whether hyperkalemia occurred during the operation as the dependent variable, the indicators with statistical differences in the univariate analysis of the hyperkalemia group and the non-hyperkalemia group were used as independent variables for Logistic analysis. The results showed that high preoperative serum potassium, low preoperative pH value, large pulse pressure, and long hemodialysis time were risk factors for Hyperkalemia during kidney transplantation. The specific results are shown in Table 2.

**Table 2:** Multivariate logistic regression analysis of hyperkalemia occurring during renal transplantation .

Factor	β	Wald*χ*^2^	*p-value*	OR(95%CI)
Preoperative hyperkalemia	5.536	16.469	<0.001	253.654 (17.501,3676.321)
Preoperative pH	−17.771	14.391	<0.001	0.000 (0.000,0.01)
Pulse pressure	0.178	18.389	<0.001	1.194 (1.101,1.295)
Hemodialysis time	1.512	14.349	<0.001	4.535 (2.074,9.916)

### 3.3 Construction of a nomogram prediction model for hyperkalemia during kidney transplantation

**Figure 1:**
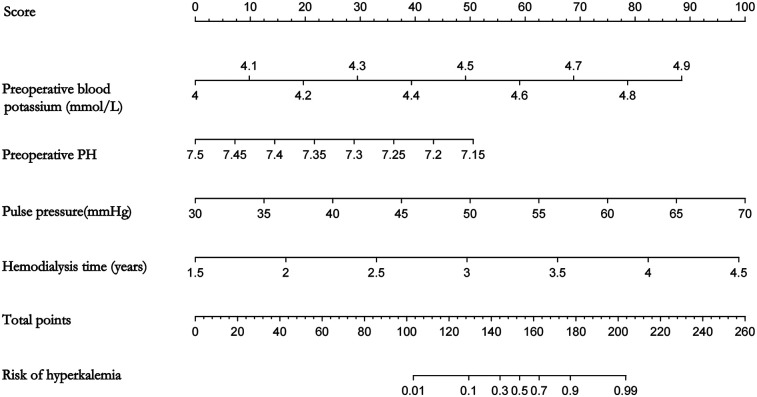
Graphic showing scales correlating different preoperative factors to risk of hyperkalemia. Factors include blood potassium levels (4 to 4.9 mmol/L), PH (7.15 to 7.5), pulse pressure (30 to 70 mmHg), and hemodialysis time (1.5 to 4.5 years). Total score ranges from 0 to 260 points, correlating to hyperkalemia risk between 0.01 and 0.99.

Based on the logistic regression analysis of four preoperative variables with statistical significance, namely preoperative serum potassium, preoperative pH value, pulse pressure, and hemodialysis time, a nomogram for predicting the risk of hyperkalemia during kidney transplantation was drawn using R software, as shown in Figure 1. The sum of the scores of each variable corresponds to the value on the risk axis, which represents the probability of hyperkalemia during kidney transplantation. The higher the total score, the higher the risk of hyperkalemia during kidney transplantation. When the predicted risk of hyperkalemia is ≥60%, the anesthesiologist will be notified to intensify intraoperative serum potassium monitoring and initiate prompt potassium-lowering interventions upon detection of serum potassium levels ≥5.5 mmol/L. Conversely, if the risk is <60%, the frequency of intraoperative potassium monitoring may be reduced. For instance, a 58-year-old male patient with a preoperative serum potassium level of 4.9 mmol/L, preoperative pH of 7.36, pulse pressure difference of 42.2 mmHg, and 3.3 years of hemodialysis history received corresponding scores of 88, 20, 30, and 55, yielding a total score of 193, which corresponds to a 95% predicted risk of developing hyperkalemia. Following reperfusion of the transplanted kidney, his serum potassium rose to 5.9 mmol/L, prompting immediate therapeutic intervention to reduce potassium levels.

### 3.4 Validation of the nomogram prediction model for hyperkalemia during kidney transplantation

**Figure 2:**
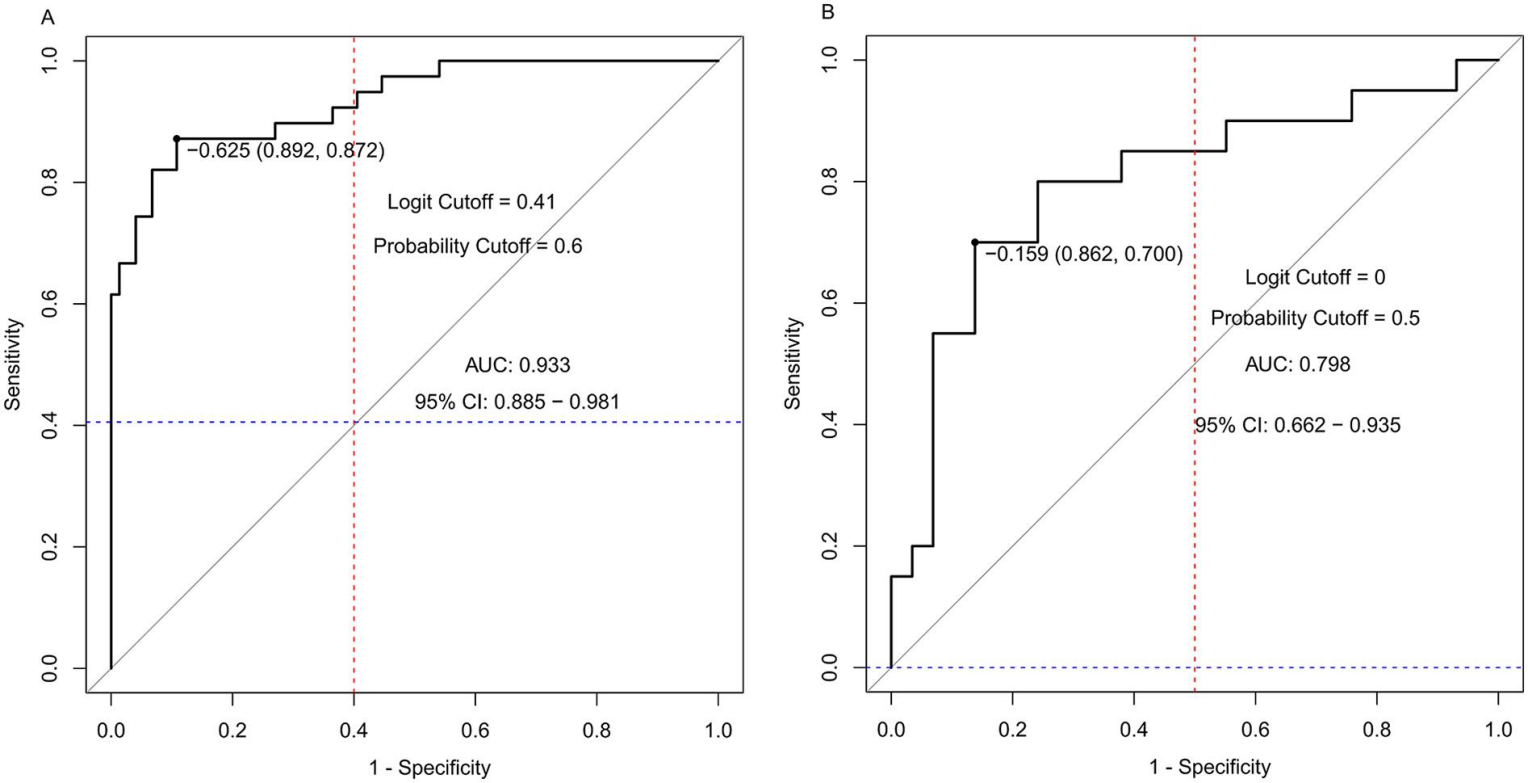
Two ROC curves labeled A and B. Graph A shows an AUC of 0.933 with a 95% confidence interval of 0. 885 to 0.981, a logit cutoff of 0.41, and a probability cutoff of 0.6. Graph B shows an AUC of 0.798 with a 95% confidence interval of 0.662 to 0.935, a logit cutoff of 0, and a probability cutoff of 0.5. Both graphs plot sensitivity versus 1-specificity with diagonal identity lines.

The ROC analysis results showed that the area under the curve of the nomogram model for predicting hyperkalemia during kidney transplantation in the training set and validation set were 0.933 (95% CI: 0.885–0.981, *P* < 0.05) and 0.798 (95% CI: 0.662–0.935, *P* < 0.05), respectively. The sensitivity, specificity, logit value, and cutoff value were 0.892, 0.872, −0.625, 0.6 and 0.862, 0.700, −0.159, 0.5, respectively (Figure 2).

**Figure 3:**
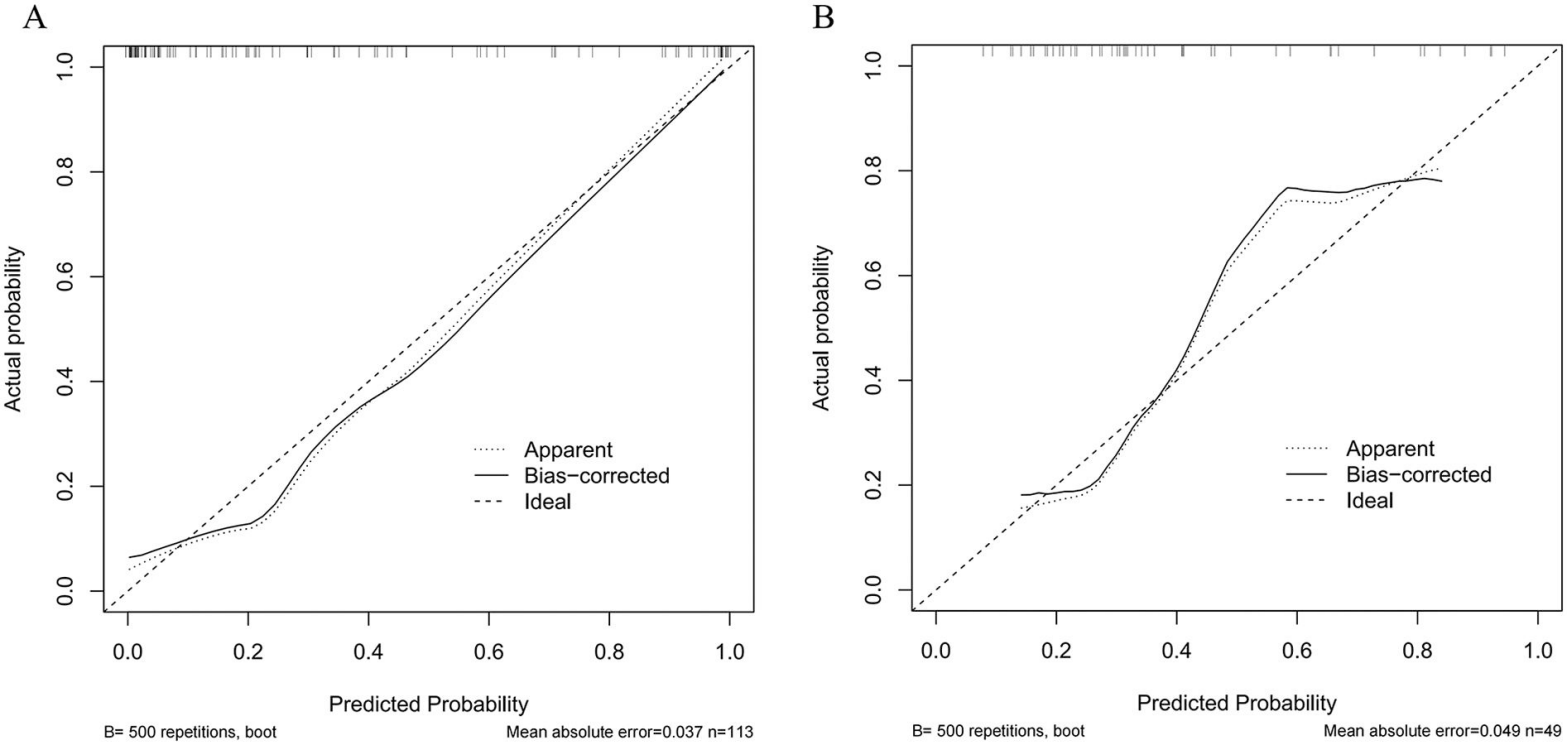
Two calibration plots, labeled A and B, compare predicted versus actual probabilities. Both plots feature three lines: dotted for apparent, solid for bias- corrected, and dashed for ideal. Plot A shows a mean absolute error of 0.037 with sample size 113, while Plot B shows a mean absolute error of 0.049 with sample size 49. Both use 500 repetitions for bootstrapping. Each graph includes a calibration curve deviating slightly from the ideal line.

The Hosmer-Lemeshow goodness-of-fit test indicated that the slope of the calibration curve in the training set was close to 1, suggesting that the nomogram prediction model fitted well. However, the slope in the validation set was worse than that in the training set, which might be due to the smaller sample size causing bias. The calibration curves were verified by using the 500 times repeated sampling method. The average absolute error in the training set was 0.037, and that in the validation set was 0.049 (Figure 3).

**Figure 4:**
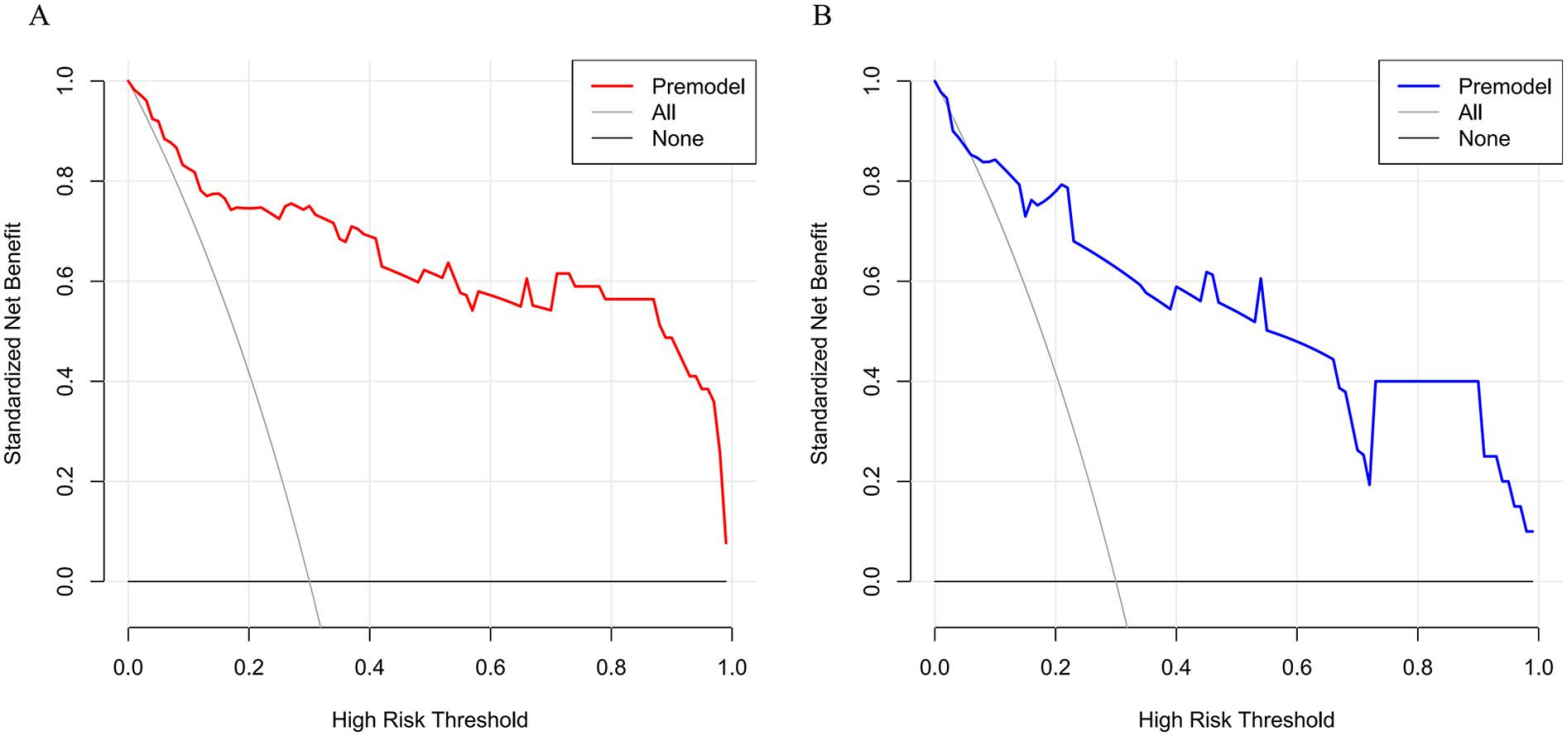
Two line graphs compare standardized net benefit against high risk threshold. Graph A displays a red “Premodel” line, showing a downward trend. Graph B shows a blue “Premodel” line with declines and stability points. Both include “All” and “None” reference lines in gray and black.

The decision curve analysis of the training set showed that when the threshold probability was 0.03–1.00, the nomogram prediction model for hyperkalemia during kidney transplantation had a high clinical net benefit rate; this was also confirmed in the validation set (Figure 4).

## 4 Discussion

Hyperkalemia is a challenging issue that needs to be addressed during the perioperative period and surgery of kidney transplantation, usually caused by multiple factors acting together (5). Its incidence rate among patients with chronic kidney disease ranges from 5% to 50% (6). A retrospective cohort study showed that the probability of hyperkalemia in patients with stage 5 end-stage renal disease is 11 times that of those without a history of end-stage renal disease (7). The severity of hyperkalemia and its related risks depend on the underlying cause and rate of potassium elevation. A short-term increase in potassium in the body may lead to cardiac arrest (8). Therefore, identifying the risk factors for hyperkalemia during kidney transplantation and constructing a nomogram model for early intervention is of great significance.

The results of this study show that preoperative hyperkalemia, low preoperative pH, large pulse pressure, and prolonged hemodialysis duration as independent risk factors for hyperkalemia during kidney transplantation. Experienced surgeons typically require 3–6 h to complete a kidney transplantation procedure. During transplantation, tissue damage and arterial clamping-induced ischemia may elevate serum potassium levels, potentially leading to hyperkalemia. For every 1 mmol/L increase in preoperative plasma potassium, the risk of requiring potassium-lowering interventions in kidney transplant recipients increases by 2.2 times post-transplantation (8). Furthermore, patients requiring potassium-lowering interventions exhibited longer hospital stays compared to those who did not (8). A low preoperative pH value may be associated with impaired renal function, particularly in CKD patients who are more prone to acidosis and potassium retention (9). In an acidotic state, the acidic intracellular environment facilitates the extrusion of potassium ions from cells into the bloodstream, thereby increasing serum potassium concentrations and inducing hyperkalemia (10). Additionally, a low pH value may stimulate increased sympathetic nerve activity, altering renal hemodynamics and further impairing the kidneys' ability to excrete potassium. Declining renal function reduces the kidneys’ potassium excretion capacity, thereby increasing the risk of hyperkalemia (11). Pulse pressure serves as a common indicator for assessing fluid volume and potassium supplementation rates. The sodium-potassium ratio grade correlates positively with systolic and diastolic blood pressure, indicating a close relationship between potassium and blood pressure (12). A large pulse pressure suggests increased fluid load, reduced urine output, and compromised renal function. An elevated pulse pressure may reflect poor systemic hemodynamics, leading to inadequate renal perfusion, subsequent renal injury, and diminished potassium excretion capacity, thus promoting hyperkalemia (13). According to the Dialysis Outcomes and Practice Patterns Study (DOPPS), 6.2%–20.0% of hemodialysis patients exhibit serum potassium levels ≥6.0 mmol/L (14). Long-term hemodialysis patients generally experience severe renal failure, accompanied by imbalances in fluid, electrolytes, and acid-base homeostasis, as well as a marked decline in renal potassium excretion capacity (15, 16). Moreover, long-term use of diuretics or renin-angiotensin-aldosterone system (RAAS) inhibitors can disrupt potassium balance and increase the risk of hyperkalemia (17, 18).

This study conducted a multivariate logistic regression analysis using data from 162 kidney transplant patients to identify independent risk factors for hyperkalemia during transplantation and develop a predictive risk model. An area under the curve (AUC) exceeding 0.9 signifies a highly effective predictive model (19, 20). In this study, the training set and validation set yielded AUC values of 0.933 (95% CI: 0.885–0.981, *P* < 0.05) and 0.798 (95% CI: 0.662–0.935, *P* < 0.05), respectively. The sensitivity and specificity were 0.892 and 0.872 for the training set, and 0.862 and 0.700 for the validation set, demonstrating that the model exhibits strong predictive performance. To further clarify the clinical significance of the model's performance metrics: The AUC values (training set: 0.933; validation set: 0.798) exceed the commonly accepted threshold of 0.7 for effective predictive models (20), with the training set AUC indicating excellent discriminative ability (AUC > 0.9) to distinguish high-risk from low-risk patients. The validation set AUC, though lower, remains clinically meaningful, suggesting the model's robustness across different patient subsets. For calibration (consistency between predicted and actual risks), the small mean absolute errors (training set: 0.037; validation set: 0.049) mean the model's predicted probabilities closely match real-world incidence, avoiding overestimation/underestimation of risk. Decision curve analysis supports utility: the high net benefit across a wide threshold range (0.03–1.00) indicates that interventions guided by the model (e.g., intensified monitoring) yield more benefits (preventing cardiac complications) than harms (unnecessary blood sampling).

In terms of clinical feasibility and integration into anesthetic protocols: The nomogram uses four preoperatively available indicators (preoperative serum potassium, pH, pulse pressure, hemodialysis duration) — all routinely collected in transplant centers, requiring no additional tests. For patients with a predicted hyperkalemia risk ≥60%, we recommend intensified intraoperative monitoring, such as frequent arterial blood gas analysis, and having potassium-lowering medications (e.g., calcium gluconate, insulin-dextrose) readily available. Intervention should be initiated promptly upon confirmation of K+ ≥5.5 mmol/L. For patients with a risk <60%, standard monitoring protocols may be sufficient. This risk-based approach aims to optimize resource allocation and enhance patient safety.

One study aimed at predicting delayed graft function (DGF) following kidney transplantation developed a nomogram utilizing donor characteristics, pre-transplant biopsy results, and machine perfusion parameters. This model achieved area under the curve (AUC) values of 0.83 in internal validation (*n* = 492) and 0.87 in external validation (*n* = 105) (21). Another investigation focusing on BK virus activation among kidney transplant recipients created a predictive model based on factors such as donor type and direct bilirubin levels, resulting in AUCs of 0.689 for the derivation cohort (*n* = 195) and 0.699 for external validation (22). In contrast, the present study is pioneering in its focus on intraoperative hyperkalemia during kidney transplantation, with an incidence rate of 36.42%. The nomogram developed herein demonstrated superior predictive performance with AUC values of 0.933 for the training set and 0.798 for the validation set. This research addresses a critical gap in intraoperative risk stratification for kidney transplantation and offers direct guidance for perioperative real-time management—an aspect not covered by the aforementioned studies.

This study has several limitations. First, the sample size is relatively small, especially the validation cohort (*n* = 49), which likely caused the AUC discrepancy (0.933 vs. 0.798) between training and validation sets and may affect model stability; future large-scale, multi-center external validation is needed. Second, its retrospective design carries bias risks—unmeasured confounders (e.g., beta-blockers, non-steroidal anti-inflammatory drugs, cardiovascular comorbidities beyond hypertension, donor characteristics) cannot be excluded despite including significant univariate variables (23). Third, excluding patients with preoperative hyperkalemia (K+ >5.0 mmol/L), though aiding intraoperative risk isolation, limits applicability to this clinically important high-risk subgroup. Additionally, there is overfitting risk due to predictor-event rate balance; while Bootstrap resampling ensured good internal calibration, the validation set's performance highlights this concern.

In summary, preoperative hyperkalemia, low preoperative pH, large pulse pressure, and prolonged dialysis duration are identified as risk factors for hyperkalemia during kidney transplantation. The nomogram model constructed based on these factors demonstrates excellent discriminative ability and calibration, offering promising predictive value.

## Data Availability

The original contributions presented in the study are included in the article/Supplementary Material, further inquiries can be directed to the corresponding authors.

## References

[1] Palmer BF Clegg DJ. Hyperkalemia treatment standard. Nephrol Dial Transplant. (2024) 39(7):1097–104. 10.1093/ndt/gfae056 38425037

[2] Grodzinsky A Goyal A Gosch K McCullough PA Fonarow GC Mebazaa A Prevalence and prognosis of hyperkalemia in patients with acute myocardial infarction. Am J Med. (2016) 129(8):858–65. 10.1016/j.amjmed.2016.03.008 27060233 PMC5031155

[3] Hunter RW Bailey MA. Hyperkalemia: pathophysiology, risk factors and consequences. Nephrol Dial Transplant. (2019) 34(Suppl 3):iii2–iii11. 10.1093/ndt/gfz206 31800080 PMC6892421

[4] Expert Group of Chinese Society of Nephrology. Expert consensus on the management of serum potassium in chronic kidney disease patients in China. Chin J Nephrol. (2020) 36(10):781–92.

[5] Sarafidis PA Blacklock R Wood E Rumjon A Simmonds S Fletcher-Rogers J Prevalence and factors associated with hyperkalemia in predialysis patients followed in a low-clearance clinic. Clin J Am Soc Nephrol. (2012) 7(8):1234–41. 10.2215/CJN.01150112 22595825 PMC3408123

[6] Einhorn LM Zhan M Hsu VD Walker LD Moen MF Seliger SL The frequency of hyperkalemia and its significance in chronic kidney disease. Arch Intern Med. (2009) 169(12):1156–62. 10.1001/archinternmed.2009.132 19546417 PMC3544306

[7] Denno DW. Physician participation in lethal injection. N Engl J Med. (2019) 380(19):1790–1. 10.1056/NEJMp1814786 31067367

[8] de Vries BCS Berger SP Bakker SJL de Borst MH de Jong MFC. Pre-Transplant plasma potassium as a potential risk factor for the need of early hyperkalaemia treatment after kidney transplantation: a cohort study. Nephron. (2021) 145(1):63–70. 10.1159/000511404 33212442 PMC7845431

[9] Babich JS Dupuis L Kalantar-Zadeh K Joshi S. Hyperkalemia and plant-based diets in chronic kidney disease. Adv Kidney Dis Health. (2023) 30(6):487–95. 10.1053/j.akdh.2023.10.001 38453264

[10] Saraf SL Derebail VK Zhang X Machado RF Gordeuk VR Lash JP Hyperkalemia and metabolic acidosis occur at a higher eGFR in sickle cell disease. Kidney360. (2022) 3(4):608–14. 10.34067/KID.0006802021 35721605 PMC9136900

[11] Ash SR Batlle D Kendrick J Oluwatosin Y Kooienga L Eudicone JM Sodium zirconium cyclosilicate in CKD, hyperkalemia, and metabolic acidosis: NEUTRALIZE randomized study. Kidney360. (2024) 5(6):812–20. 10.34067/KID.0000000000000446 38622759 PMC11219110

[12] Yoon Y Son M. Association between blood pressure control in hypertension and urine sodium to potassium ratio: from the Korea national health and nutrition examination survey (2016–2021). PLoS One. (2024) 19(11):e0314531. 10.1371/journal.pone.0314531 39591407 PMC11594522

[13] Gupta AA Self M Mueller M Wardi G Tainter C. Dispelling myths and misconceptions about the treatment of acute hyperkalemia. Am J Emerg Med. (2022) 52:85–91. 10.1016/j.ajem.2021.11.030 34890894

[14] Saran R Bragg-Gresham JL Rayner HC Goodkin DA Keen ML Van Dijk PC Nonadherence in hemodialysis: associations with mortality, hospitalization, and practice patterns in the DOPPS. Kidney Int. (2003) 64(1):254–62. 10.1046/j.1523-1755.2003.00064.x 12787417

[15] Sampani E Theodorakopoulou M Iatridi F Sarafidis P. Hyperkalemia in chronic kidney disease: a focus on potassium lowering pharmacotherapy. Expert Opin Pharmacother. (2023) 24(16):1775–89. 10.1080/14656566.2023.2245756 37545002

[16] St-Jules DE Fouque D. Etiology-based dietary approach for managing hyperkalemia in people with chronic kidney disease. Nutr Rev. (2022) 80(11):2198–205. 10.1093/nutrit/nuac026 35482610

[17] Zhao X Hou FF Liang X Ni Z Chen X Chen Y High facility-level serum potassium variability associated with mortality in hemodialysis patients: results from Chinese dialysis outcomes and practice patterns study (DOPPS). Ren Fail. (2023) 45(1):2211157. 10.1080/0886022X.2023.2211157 37293774 PMC10259339

[18] De Nicola L Ferraro PM Montagnani A Pontremoli R Dentali F Sesti G. Recommendations for the management of hyperkalemia in patients receiving renin-angiotensin-aldosterone system inhibitors. Intern Emerg Med. (2024) 19(2):295–306. 10.1007/s11739-023-03427-0 37775712 PMC10954964

[19] Spinowitz BS Fishbane S Pergola PE Roger SD Lerma EV Butler J Sodium zirconium cyclosilicate among individuals with hyperkalemia: a 12-month phase 3 study. Clin J Am Soc Nephrol. (2019) 14(6):798–809. 10.2215/CJN.12651018 31110051 PMC6556727

[20] Babich JS Dupuis L Kalantar-Zadeh K Joshi S. A nomogram to predict hyperkalemia in patients with hemodialysis: a retrospective cohort study. BMC Nephrol. (2022) 23(1):351. 10.1186/s12882-022-02976-4 36319967 PMC9628065

[21] Li M Hu X Li Y Chen G Ding CG Tian X Development and validation of a novel nomogram model for predicting delayed graft function in deceased donor kidney transplantation based on pre-transplant biopsies. BMC Nephrol. (2024) 25(1):138. 10.1186/s12882-024-03557-3 38641807 PMC11031976

[22] Wang J Li J Chen Z Xu M Yang C Rong R A nomogram for predicting BK virus activation in kidney transplantation recipients using clinical risk factors. Front Med (Lausanne). (2022) 9:770699. 10.3389/fmed.2022.770699 35223891 PMC8866320

[23] Palmer BF Clegg DJ. Managing hyperkalemia to enable guideline-recommended dosing of renin-angiotensin-aldosterone system inhibitors. Am J Kidney Dis. (2022) 80(2):158–60. 10.1053/j.ajkd.2022.02.012 35461743

